# The Indigo System in Acute Lower-Limb Malperfusion (INDIAN) Registry: Protocol

**DOI:** 10.2196/resprot.9972

**Published:** 2019-03-14

**Authors:** Gianmarco de Donato, Edoardo Pasqui, Giovanni Giannace, Francesco Setacci, Domenico Benevento, Giancarlo Palasciano, Carlo Setacci, INDIAN Registry Collaborators

**Affiliations:** 1 Department of Medicine, Surgery and Neuroscience University of Siena Siena Italy; 2 Istituto di Ricovero e Cura a Carattere Scientifico (IRCCS) Multimedica Milan Italy

**Keywords:** acute limb ischemia, endovascular, mechanical thrombectomy

## Abstract

**Background:**

Acute lower limb ischemia (ALLI) poses a major threat to limb survival. For many years, surgical thromboembolectomy was the mainstay of treatment. Recent years have brought an endovascular revolution to the management of ALLI. It seems that the newly designed endovascular thrombectomy devices may shift treatment recommendations toward endovascular options. This protocol study aims to collect evidence supporting the latest hypothesis.

**Objective:**

The devices under investigation are the Penumbra/Indigo Systems (Penumbra Inc). The objective of this clinical investigation is to evaluate, in a controlled setting, the early safety and effectiveness of the devices and to define the optimal technique for the use of these systems in patients with confirmed peripheral acute occlusions.

**Methods:**

This study will be an interventional prospective trial of patients with a diagnosis of ALLI treated with Penumbra/Indigo devices. This project is intended to be a national platform where every physician invited to participate could register his or her own data procedure. The primary outcome is the technical success of thromboaspiration with the Indigo System. Assessment of vessel patency will be recorded using the Thrombolysis in Myocardial Infarction (TIMI) score classifications before and after use of the device. Clinical success at follow-up is defined as an improvement of Rutherford classification at 1-month follow-up of one class or more as compared to the preprocedure Rutherford classification. Secondary endpoints include the following: (1) safety rate at discharge, defined as the absence of any serious adverse events; (2) primary patency at 1 month, defined as a target lesion without a hemodynamically significant stenosis or reocclusion on duplex ultrasound (>50%) and without target lesion reintervention within 1 month; and (3) limb salvage at 1 month.

**Results:**

The study is currently in the recruitment phase and the final patient is expected to be treated by the end of March 2019. A total of 150 patients will be recruited. Analyses will focus on primary and secondary endpoints.

**Conclusions:**

These new endovascular thrombectomy devices that are specifically designed for peripheral intervention in this difficult set of patients, as those under investigation in the proposed registry, may offer improved clinical outcomes with lower rates of major systemic and local complications. Following completion of this study, it is expected that the value of the Indigo Thrombectomy System in the treatment of ALLI will be better defined. As a result, a shift of treatment recommendations toward endovascular options may be observed in the near future.

**International Registered Report Identifier (IRRID):**

DERR1-10.2196/9972

## Introduction

### Background

Acute lower limb ischemia (ALLI) poses a major threat to limb survival. For many years, surgical thromboembolectomy was the mainstay of treatment. Recent years have brought an endovascular revolution to the management of ALLI. A wide range of endovascular procedures can nowadays be employed, providing results at least as good as the traditional surgical approach.

Since the first successful embolectomy performed by Georges Labey in 1911, the treatment goal has been to restore adequate blood supply to the extremity as soon as possible [1]. After introduction of the balloon catheter by Fogarty in the mid-1960s, surgical thromboembolectomy was considered the gold standard of treatment for many years [2].

Minimally invasive techniques emerged at the beginning of the 1970s when Dotter et al first introduced the idea of clot lysis in the treatment of ALLI. After discouraging first experiences with general thrombolysis, the concept of percutaneous, catheter-directed thrombolysis (CDT) emerged [3]. Since then, many new techniques utilizing sophisticated equipment have been developed to improve treatment results and reduce complications.

Significant changes in the treatment of ALLI have been observed in recent years. Although surgery still plays an important role, endovascular techniques are gaining a more prominent role in this difficult set of patients. It seems that the newly designed endovascular thrombectomy devices may shift treatment recommendations toward endovascular options.

This protocol study aims to collect evidence supporting the latest hypothesis.

### Fogarty Catheter Thromboembolectomy

Fogarty thromboembolectomy offers several advantages. In cases of limb-threatening ischemia due to large-vessel embolic occlusion, it can be promptly performed via femoral approach using local anesthesia.

The operation is relatively easy to perform even by less-experienced operators. It allows immediate therapeutic heparinization, which is considered to improve outcomes [3]. A successful operation results in an instant perfusion improvement in the ischemic limb.

In cases of below-the-knee embolism and/or arterial thrombosis, the surgical treatment is more complicated. Precise thromboembolectomy via inguinal incision is very difficult using a standard Fogarty catheter (ie, low torquability).

The walls of peripheral vessels are fragile and prone to vasospasm. Additionally, back-bleeding is not a reliable indicator of adequate thromboembolectomy [4]. Surgical approach to the popliteal artery requires general or spinal anesthesia, extensive dissection, a more experienced operator and, often, patch angioplasty. The drawbacks of a “blind” popliteal embolectomy may be reduced by using an intraoperative C-arm and application of a modified Fogarty catheter with an additional channel for guidewire navigation. Using an intraoperative C-arm, targeted thrombectomy is feasible and provides improved results [5].

Sometimes, when adequate thrombus removal cannot be achieved, especially in patients with thrombosed popliteal aneurysm or small vessel thrombosis, intra-arterial injection of a thrombolytic agent at the time of revascularization or other hybrid solutions are advocated [6,7].

Although simple thromboembolectomy still plays a significant role in the treatment of ALLI, fundamental changes in the patient population have occurred in recent decades.

Decreasing incidence of rheumatic heart disease and widespread anticoagulation therapy in patients with cardiac arrhythmias has largely reduced the frequency of peripheral embolism. Nowadays, in the majority of patients, the symptoms of ALLI are the result of either advanced atherosclerosis, complications of previous vascular procedures, or peripheral aneurysms. Surgical treatment in such cases is much more demanding and often requires additional patch angioplasty or a vascular bypass.

Moreover, surgical treatment of ALLI, although effective, has significant drawbacks. Residual thrombus, propagation of thrombi, chronic atherosclerotic disease, and vessel injuries secondary to Fogarty catheter passage may limit the clinical success rate.

### Fibrinolysis

The concept of thrombolysis was introduced by Dotter in the early 1970s. Initial experience with systemic thrombolysis was not encouraging. The treatment was ineffective and burdened with a high rate of bleeding complications. Further development of thrombolysis resulted in catheter direct thrombolysis, which is currently widely utilized in clinical settings. Nowadays, although some contraindications exist, the majority of patients with less severe ischemia (ie, classes 1, 2a, and sometimes 2b, according to Rutherford’s classification) [8] can be offered CDT.

Bleeding is considered to be the most formidable complication of thrombolysis, especially in older patients and when the therapy exceeds 48 hours. Reported data suggest that approximately 5%-15% of treated patients suffer major bleeding that requires intervention. Intracranial bleeding occurs in less than 1% of patients; however, it is lethal in most cases.

Distal embolization is another serious complication of CDT. The reported frequencies vary between 0% and 37% of treated patients [9,10]. Uncontrolled disruption of the distal portion of the thrombus may pose a significant threat to the limb survival. Small thrombi tend to migrate to peripheral arteries, in which case treatment—surgical and/or endovascular—is difficult and the result is often unsatisfactory. Some authors recommend distal filter placement during CDT in order to reduce the risk [11]. Such a protocol seems effective but at the cost of possible filter-related complications and significant economic burden.

### Mechanical Aspiration Thrombectomy

Mechanical thrombectomy devices are the latest advancement in the field of ALLI treatment. Aspiration thrombectomy is a method of thrombus evacuation in patients with ALLI with some potential advantages over thrombolysis: prompt reperfusion (ie, minutes, not hours) and feasibility in patients with contraindications to thrombolysis and nonthrombotic material removal (ie, peripheral atheromatous emboli after endovascular procedures). As opposed to rheolytic thrombectomy, it does not induce hemolysis. It may be utilized in patients with contraindications to thrombolysis (ie, hepatic failure, recent surgery, trauma, or a neurovascular accident).

### The Indigo System

The devices under investigation are the Penumbra/Indigo Systems (Penumbra Inc). At the start of the study, Indigo catheters 8 (straight/torq/Xtorq tip [STR/TORQ/XTORQ]), 6, 5, and 3 and Indigo Separators had obtained European Conformity (CE)-approval and are all indicated for the removal of fresh, soft emboli and thrombi from vessels of the peripheral arterial and venous systems.

The Indigo System’s aspiration is generated by an external vacuum generator. The system works on the over-the-wire platform. The Indigo device is a polymer-covered, nitinol-strengthened 6 French gauge (Fr) catheter with a suction port at the tip of the device.

During the procedure, the specially designed occlusion catheter is first located proximally to the lesion. It also works as a guiding catheter for the suction unit. The suction catheter is advanced to the level just beneath the thrombus. A separator with an olive-shaped soft tip is advanced and withdrawn several times through the thrombus to disrupt it and facilitate aspiration.

The objective of this clinical investigation is to evaluate, in a controlled setting, the early safety and effectiveness of the Penumbra/Indigo aspiration thrombectomy Systems (Penumbra Inc) and to define the optimal technique for the use of these systems in patients with confirmed peripheral acute occlusions.

## Methods

### Patient Population and Setting

The Indigo System in Acute Lower-Limb Malperfusion (INDIAN) Registry was intended as a national platform where every physician could register his or her own data procedure.

A total of 150 patients suffering from acute lower limb malperfusion will be recruited in order to prove safety and efficacy of the Indigo System. All participating centers have extensive experience in this kind of disease. The ethical committee of each hospital was informed of the nonexperimental design of the protocol, considering that the devices under investigation have CE mark approval, and endorsed the project.

The anticipated duration of this clinical investigation is approximately 13 months. It is estimated that the inclusion period will be 12 months. The follow-up period is set to be 1 month. The actual start date of the investigation was September 2017, the estimated primary completion date is March 2019, and the estimated study completion date is May 2019.

Patients will be selected based on the investigator’s assessment and evaluation of the underlying disease. Each patient’s medical condition should be stable, with no underlying medical condition that would prevent them from performing the required testing or from completing the study.

Patients should be geographically stable, willing and able to cooperate in this clinical study, and remain available for midterm follow-up. Patients who do not wish to participate in this study can obtain any other standard commercially available device therapy. Refusal to participate in this study will in no way affect their care at the institution. Inclusion and exclusion criteria are listed in Textbox 1.

**Inclusion and exclusion criteria for the study.**

Inclusion criteria:

- Patient presenting with an acute occlusion of lower limb arteries; thrombosis no longer than 14 days
- Patient presenting a score from 1 to 2b following Rutherford classification for acute limb ischemia
- Patient is willing to comply with specified follow-up evaluations at the specified times for the duration of the study
- Patient is >18 years old
- Patient, or their legal representative, understands the nature of the procedure and provides written informed consent, prior to enrollment in the study
- Patient is eligible for treatment with the Indigo System (Penumbra Inc)

Exclusion criteria:

- Patient has an estimated time of intraluminal thrombus of >14 days
- Patient refuses treatment
- Patient for whom antiplatelet therapy, anticoagulants, or thrombolytic drugs are contraindicated
- Patient with a history of prior life-threatening contrast medium reaction
- Patient has life expectancy of less than 6 months
- Patient is considered to be hemodynamically unstable at onset of procedure

### Endpoints

The primary endpoint of the study is the technical success of the thromboaspiration with the Indigo System. Assessment of vessel patency will be recorded using the Thrombolysis in Myocardial Infarction (TIMI) score classification before and after the use of the device [12].

The following secondary endpoints will be assessed:

1. Clinical success at 1-month follow-up defined as an improvement of Rutherford classification of one class or more as compared to the preprocedure Rutherford classification.
2. Safety rate at discharge defined as absence of any serious adverse events, such as any clinical event that is fatal, life-threatening, or judged to be severe by the investigator, that resulted in persistent or significant disability.
3. Primary patency at 1 month, defined as a target lesion without a hemodynamically significant stenosis or reocclusion on duplex ultrasound (>50%) and without target lesion reintervention within 1 month.

### Data Collection and Analysis

Patient data will be captured electronically using a cloud platform accessible to all investigators.

Descriptive data summaries will be used to present and summarize the collected data. For categorical variables (eg, gender), frequency distributions and cross tabulations will be given. For numeric variables (eg, patient age), minimum, maximum, mean, median, and standard deviation will be calculated. For all variables, a 95% confidence interval for the relevant parameters of the underlying distribution will be calculated. For all time-dependent events, life tables will be calculated using the Kaplan Meier estimate method for a period starting on the date of the procedure up to and including the 24-month follow-up visit. Stratification to preprocedural risk factors, Rutherford, and lesion criteria will be performed and the log rank test will be used to compare between the different outcomes; associated *P* values <.05 will be defined as significant.

Additionally, all peri- and postprocedural complications (<24 hours) will be evaluated and documented.

### Patient Confidentiality

All information and data concerning patients or their participation in this clinical investigation will be considered confidential. Only authorized personnel will have access to these confidential files. Authorized personnel of health authorities will have the right to inspect and copy all records pertinent to this clinical investigation. All data used in the analysis and reporting of this clinical investigation will be without identifiable reference to a specific patient name.

## Results

Patient enrollment started in October 2017. It is anticipated that 150 patients will be recruited to the study. The final patient is expected to be treated by the end of March 2019 and the estimated study completion date is May 2019. Data will be analyzed by the coordinating center and results will be shared with each investigating center.

## Discussion

After the invention of the balloon catheter by Fogarty in 1963, surgical thromboembolectomy was considered the gold standard of treatment for many years in patients with ALLI. Still, ALLI is a dramatic event, carrying a significant risk of amputation and high perioperative morbidity and mortality. Therefore, the need for continued innovation in this area led to innovative percutaneous approaches. In the 1970s, Dotter first introduced the idea of clot lysis in the treatment of ALLI, then modified to the catheter-directed thrombolysis [2]. Since then, many new endovascular procedures utilizing sophisticated equipment have been developed to improve treatment results and reduce complications.

Currently, the majority of ALLI (approximately 70%) is arterial thrombosis, which generally occurs in the setting of pre-existing vascular lesion. This condition is very common in patients with diabetes. Clinical presentation in the case of thrombosis on atherosclerotic stenosis—so-called “acute on chronic ischemia”—may be less severe. However, treatment is generally more challenging than for ALLI due to embolism, considering the complexity in device trackability through the diseased vessels, potential vessel injury, incomplete revascularization, and need of correction of underlying vascular lesions.

Although surgery is still a significant treatment option, especially for ALLI due to embolism, endovascular techniques are gaining a more prominent role in the case of acute on chronic ischemia. Improved clinical outcomes, associated with lower rates of major complication coming from the application of newly designed endovascular thrombectomy devices in this difficult set of patients, may shift treatment recommendations toward endovascular options. In this scenario, the rapid development of technology allows physicians to choose between different approaches, techniques, and devices.

The Indigo System promotes active thrombectomy using a vacuum pump that generates substantial suction, enabling aspiration of clots of varying sizes and lengths. The device has three components: aspiration catheter, separator, and pump. The system does not contain any rotational components; therefore, the risk of vessel injury is truly minimized. The Indigo System represents a last-generation system for thromboembolic disease, being designed specifically to address the limitations of conventional technology.

Since 2005, the Penumbra System became available in Europe and the United States for the revascularization of occluded intracranial vessels in patients with acute ischemic stroke. By demonstrating that the system is safe and effective in the neurovasculature and by providing high rates of complete intracranial vessel revascularization, the Penumbra System has become the market leader in stroke treatment. Consequently, physicians who were familiar with the Penumbra System for stroke care started using it in the peripheral vasculature for acute thrombotic and embolic events. Lesions that were previously inaccessible with conventional technology were then treated by these atraumatic, ultraflexible neurovascular devices. In 2014, Penumbra launched the Indigo System specifically for this application, redefining below-the-knee mechanical thrombectomy.

Although there are some very promising case report experiences, clinical data with this thrombectomy device in patients with ALLI is still limited. An ongoing trial, called the Penumbra and Indigo Systems for Mechanical Thrombectomy in the Periphery (PRISM) trial [13], has been designed to evaluate safety and efficacy of the Indigo thrombectomy catheter. The examined population consists of ALLI patients with thrombolysis failure. Partial results were recently published, showing promising results with safe and effective mechanical thromboembolectomy in the peripheral arterial vasculature (ie, they showed a technical success rate of 86.4%). These results were reached across a broad range of clinical applications, including acute ischemia, removal of emboli that occurred during other endovascular procedures, and after failed thrombolysis.

In conclusion, suggestive modifications in the treatment of ALLI have been proposed in recent years. While surgery still represents a significant treatment option, especially for ALLI due to embolism, endovascular techniques are acquiring a more prominent role in the case of acute on chronic ischemia.

Various mechanical endovascular systems for thrombus removal have been investigated over the last 15 years. Most of them have partially failed to be successful or have been associated with undesirable complication rates.

New endovascular thrombectomy devices specifically designed for peripheral intervention in this difficult set of patients, as the one under investigation in the proposed registry, may offer improved clinical outcomes with lower rates of major systemic and local complications.

After completion of this study, data analysis from the INDIAN Registry may clarify the value of the Indigo Thrombectomy System in the treatment of ALLI. As a result, a shift of treatment recommendations toward endovascular options may be observed in the near future.

## Abbreviations

ALLI: acute lower limb ischemia
CDT: catheter-directed thrombolysis
CE: European Conformity
Fr: French gauge
INDIAN: Indigo System in Acute Lower-Limb Malperfusion
PRISM: Penumbra and Indigo Systems for Mechanical Thrombectomy in the Periphery
STR/TORQ/XTORQ: straight/torq/Xtorq tip
TIMI: Thrombolysis in Myocardial Infarction
